# Postprandial Effects of Salmon Fishmeal and Whey on Metabolic Markers in Serum and Gene Expression in Liver Cells

**DOI:** 10.3390/nu14081593

**Published:** 2022-04-12

**Authors:** Marit Hjorth, Natalia M. Galigniana, Ola Ween, Stine M. Ulven, Kirsten B. Holven, Knut Tomas Dalen, Thomas Sæther

**Affiliations:** 1 Department of Nutrition, Institute of Basic Medical Sciences, University of Oslo, Sognsvannsveien 9, Domus Medica, 0372 Oslo, Norway; marit.hjorth@medisin.uio.no (M.H.); smulven@medisin.uio.no (S.M.U.); k.b.holven@medisin.uio.no (K.B.H.); k.t.dalen@medisin.uio.no (K.T.D.); 2 Department of Molecular Medicine, Institute of Basic Medical Sciences, University of Oslo, Sognsvannsveien 9, Domus Medica, 0372 Oslo, Norway; n.m.galigniana@medisin.uio.no; 3 Møreforskning AS, Borgundvegen 340, 6009 Ålesund, Norway; ola.ween@moreforsk.no; 4 Norwegian National Advisory Unit on Familial Hypercholesterolemia, Oslo University Hospital, P.O. Box 4959, Nydalen, 0424 Oslo, Norway

**Keywords:** fishmeal, whey, fish protein, postprandial, transcriptomics

## Abstract

Fish is considered an important part of a healthy diet, in part due to the content of long chain omega-3 fatty acids. However, both lean and fatty fish have beneficial health effects, suggesting that micronutrients and proteins may play a role. In a randomised, controlled, cross-over trial, five healthy male participants consumed 5.2 g of protein from either salmon fishmeal or whey. Blood samples were taken before and 30 and 60 min after intake. The concentration of glucose, lipids, hormones and metabolites, including 28 different amino acids and derivatives, were measured in serum or plasma. Cultured HepG2 cells were incubated with or without serum from the participants, and transcriptomic profiling was performed using RNA sequencing. The ingestion of both salmon fishmeal and whey reduced the glucose and triglyceride levels in serum. Protein intake, independent of the source, increased the concentration of 22 amino acids and derivatives in serum. Fishmeal increased the concentration of arginine, methionine, serine, glycine, cystathionine and 2-aminobutyric acid more than whey did. Incubation with postprandial serum resulted in large transcriptomic alterations in serum-fasted HepG2 cells, with the differential expression of >4500 protein coding genes. However, when comparing cells cultivated in fasting serum to postprandial serum after the ingestion of fishmeal and whey, we did not detect any differentially regulated genes, neither with respect to the protein source nor with respect to the time after the meal. The comparable nutrigenomic effects of fishmeal and whey do not change the relevance of fish by-products as an alternative food source.

## 1. Introduction

The world’s population is expected to grow to almost 10 billion by 2050, which will create higher demands on food production systems [1]. A major challenge for the future is to improve agricultural and aquacultural sustainability and efficiency. As a part of this, it is necessary to explore the potential use of alternative food resources at our disposal. One such resource is by-products from the fish farming industry, like fish viscera, backbone, skin, fins, and head [2,3].

In Norway, one of the world’s largest fishery nations, 27% of all caught and farmed fish and seafood is characterized as by-products [4], of which a majority is used for animal feed production [4,5]. However, by-products from fish contain high amounts of proteins, and may serve as a significant source of human nutrition [2,3].

Fish is considered an important part of a healthy diet, and a public health recommendation is to increase dietary intake of fish to two to three servings per week [6,7]. The beneficial health effects of fish are in part due to the content of long chain omega-3 fatty acids. However, both lean and fatty fish have beneficial health effects [8] suggesting that also other components in fish are contributing factors. As an example, fish also contains vitamin D, iodine, selenium and taurine. Furthermore, certain studies have reported beneficial effects of fish protein or bioactive peptides on cardiometabolic risk factors [9]. In a human intervention study, dietary cod fillet protein improved insulin sensitivity [10].

Another study demonstrated the potential effects of a codfish-protein supplement on glucose tolerance and LDL cholesterol [11]. However, the data are inconsistent [12,13]. We recently reported no effect of 8 weeks of supplementation with salmon protein on glucose tolerance or serum lipids [13]. Lastly, several studies on rodents have suggested that salmon protein hydrolysates may improve postprandial glucose regulation, slow body weight gain, lower plasma cholesterol and influence the hepatic lipid metabolism [14,15,16]. Few, if any of these studies have addressed the transcriptomic changes that might occur in active metabolic tissues, such as the liver, as a result of the fish-protein supplementation.

In this study, we aimed to investigate the postprandial effects of protein-rich fishmeal produced from salmon by-products compared to the effects of whey on the gene expression in cultured liver cells. To this end, we conducted a randomised, controlled cross-over trial on five healthy male participants, characterized the nutrient composition of fishmeal and whey, and analysed the serum concentration of metabolic markers and amino acids after the intake of these supplements. Finally, HepG2 cells were incubated with postprandial serum from the participants, and the gene expression profiles were assessed using RNA sequencing.

## 2. Material and Methods

### 2.1. Postprandial Study

The study (ClinicalTrials.gov Identifier: NCT04078958) was conducted according to the Declaration of Helsinki and was approved by the Regional Ethics Committee for Medical Research in South East Norway. All participants provided written, informed consent. Five male participants were recruited to the study. The inclusion criteria were age ≥ 20 years, and a BMI of 18.5–24.9. Exclusion criteria included high blood pressure, known diabetes and intolerance or allergies to dairy or fish. All participants had blood levels of ferritin, aspartate aminotransferase (AST), alanine aminotransferase (ALT) and gamma-glutamyl transferase (GGT) within normal reference ranges.

**Figure 1:**
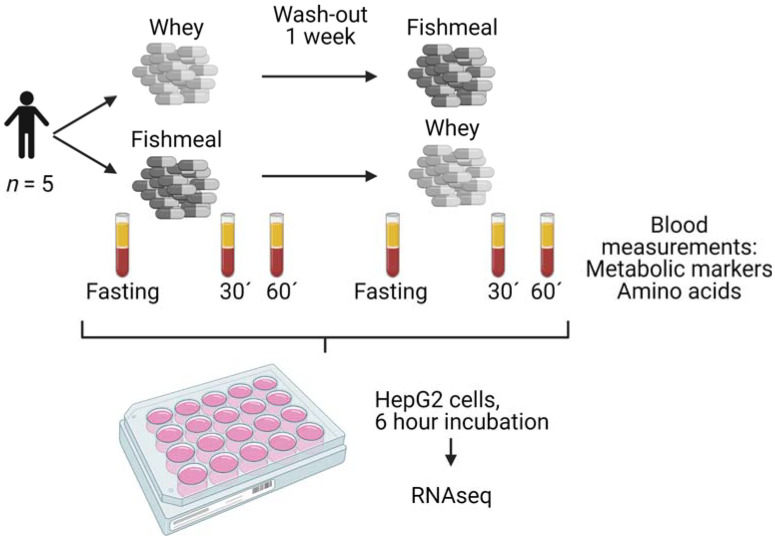
Experimental set-up. The study was a randomised, controlled, cross-over study on five healthy male participants. The participants were given 30 capsules containing a total of 5.2 g protein from salmon fishmeal and whey, separated by a 1 week washout period. Blood samples were taken before (fasting) and 30 and 60 min after intake. The effects of fishmeal and whey on metabolic markers and serum amino acid concentrations were measured. Cultured HepG2 cells were incubated for 6 h in serum-free medium or with 20% human serum from all time points. The response in gene expression was measured with RNA sequencing. The figure was created with BioRender.com.

The study was designed as a single-blinded, randomised, controlled, cross-over trial. The participants completed two postprandial tests of fishmeal and whey, separated by a 1 week wash-out period (Figure 1). Participants arrived fasting (8–12 h) in the morning of each test and ingested 30 capsules, containing a total of 5.2 g of protein from either salmon fishmeal or whey, with 0.5 L of water.

The day before the test, they refrained from consuming dietary supplements, fish and more than two portions of dairy products. The test dose was chosen based on assessments performed in our recent intervention study [13], balancing the protein amount, capsule size and capsule number against compliance and the amount of excipients. We found that 5.2 g of salmon protein equals approximately the amount found in 25 g of salmon fillet, which would equal one serving a week (175 g/week) [13].

Venous blood samples were taken before (fasting) and 30 and 60 min after the intake of fishmeal or whey (Figure 1). The participants were asked to refrain from hard physical activity during the sampling period. Serum and plasma were obtained from silica gel tubes and K2EDTA tubes, respectively (Becton, Dickinson and Company, Franklin Lakes, NJ, USA). For plasma glucagon-like peptide (GLP1) measurements, K2EDTA tubes were treated with dipeptidyl peptidase IV inhibitors (Sigma-Aldrich, St. Louis, MO, USA).

High quality salmon fishmeal was produced from the by-products of farmed Atlantic salmon and consisted of approximately *w*/*w* 50% viscera, 25% backbone, and 25% head (Mowi ASA, Bergen, Norway). Whey protein powder (WPC 80) was produced by Tine SA (Oslo, Norway). The nutritional content, including amino acids, vitamins and minerals, was analysed by Eurofins Food & Feed Testing Norway AS (Moss, Norway).

The nutritional content is displayed in Table 1 and Table 2 and in Supplementary Materials Table S1. Fishmeal and whey were packaged (Optipharma AS, Drøbak, Norway) in transparent, bovine gelatine capsules (96 mg gelatine/capsule, ACG Europe Ltd., Reading, UK), with microcrystalline cellulose (91–240 mg/capsule), antioxidants (tocopherols and rosemary extract) and excipients (magnesium stearate at 5 mg/capsule, tricalsium phosphate at 5 mg/capsule and silicon dioxide at 2.5 mg/capsule). A single dose consisted of 30 capsules, providing 5.2 g of protein, which was equivalent to 6.75 g of whey or 7.50 g of fishmeal.

### 2.2. Analyses in Serum and Plasma

The serum concentrations of glucose, insulin, triglycerides, total cholesterol, AST, ALT, ferritin, GGT and microCRP were measured by standard methods at an accredited routine laboratory (Fürst Medical Laboratory, Oslo, Norway). The plasma GLP1 was measured with ELISA (cat# 81506, Crystal Chem, Elk Grove, IL, USA).

The serum amino acid concentrations were measured by high-performance liquid chromatography–tandem mass spectrometry (HPLC-MS/MS), as previously described [17]. Chromatographic separation was performed on a Phenomenex Kinetex Core Shell C18 system (100 × 4.6 mm, 2.6 μm) with an aqueous solution containing formic acid (0.5%), heptafluorobutyric acid (0.3%) and acetonitrile. Linear calibration curves of the peak area ratios of analytes and internal standards were used for quantification.

### 2.3. Cell Culture Experiments

HepG2 cells were grown in DMEM (Cat# D6546, Sigma-Aldrich) supplemented with 10% FBS (Sigma-Aldrich). For experiments, the cells were kept in serum-free medium overnight, and then incubated for 6 h in DMEM without serum (*n* = 6) or in DMEM with 20% human serum from the postprandial study (Figure 1; *n* = 5 for fishmeal and whey, after fasting and 30 and 60 min after consumption). The total RNA was isolated with a NucleoSpin RNA mini kit (Machery-Nagel, Düren, Germany), according to the manufacturer’s instructions.

### 2.4. cDNA Synthesis and Quantitative RT-PCR

Isolated RNA (500 ng) was reverse transcribed into cDNA using MultiScribe Reverse Transcriptase (Cat# 4311235, Thermo Fisher Scientific, Waltham, MA, USA) and random hexamer primers. RT-qPCR was performed with 2 µL of a 1:5 dilution of the cDNA synthesis reaction using Kapa SYBR FAST qPCR Master Mix (Cat# KK4600, KapaBiosystems, Roche, Basel, Switzerland) on a Bio-Rad CFX96 Touch™. The assay primers were designed with Primer-BLAST software (NCBI, Bethesda, MD, USA) [18]. The gene expression was calculated using the 2-ΔΔCT method and normalized against the expression of TATA-binding protein (*TBP*). All primer pairs showed an efficiency of 90–110% at R^2^ > 0.98. The primer sequences are listed in Supplementary Materials Figure S2.

### 2.5. RNA Sequencing and Read Counting

The total RNA integrity was analysed with a Bioanalyzer RNA 6000 Nano Kit (Agilent Technologies, Santa Clara, CA, USA). All RNA samples had RIN values >9.6. RNA samples were sequenced at the Norwegian Sequencing Centre (Oslo University Hospital, Ullevål, Oslo, Norway). The RNA samples were subjected to Strand-Specific TruSeqTM mRNA-seq library preparation, and 50 bp paired-end reads were sequenced on an Illumina NovaSeq 600 instrument.

BBDuk (BBMap v34.56) was used to remove/trim low-quality reads and adapter sequences [19]. Reads were then mapped to a human reference genome (ENSEMBL release 101, GRCh38.101) using HiSat2 v.1.2.1 [20]. Read counting was done with FeatureCounts v1.4.6-p1 [21]. The average read count was 40 million/sample. The RNA sequencing data reported in this article is available at NCBI GEO (accession number GSE178584).

### 2.6. Data Analysis

For analyses of RNA sequencing data, we used R v4.0.3 software. Differential expression analyses were done in the EdgeR package using a glmQLFTest, with both paired or unpaired design [22]. A false discovery rate (fdr) < 0.05 was used as the threshold for statistical significance. PCA plots were generated from log_2_-transformed transcripts per million (TPM) expression values, using the prcomp function in R. Gene ontology analyses were done in the clusterProfiler package with KEGG pathway annotations [23] using the default parameters: organism = hsa, keyType = kegg, pvalueCutoff = 0.05, pAdjustMethod = BH, minGSSize = 15, maxGSSize = 500 and qvalueCutoff = 0.25. Other analyses were done in GraphPad Prism version 8, with methods for statistical testing listed in the figure and table legends.

## 3. Results and Discussion

To compare the postprandial effects of salmon fishmeal to whey, we started by characterizing the nutritional content of the two protein sources before we conducted a small postprandial study with five healthy males. The participants were between 33 and 45 years old and had a BMI of 21.5 to 24.0 kg/m^2^. Only males were included in the study to reduce the inter-donor variation. The concentration of metabolites and hormones were measured in serum or plasma sampled before (fasting), and 30 and 60 min after the meal. HepG2 cells were then stimulated with the same serum and analysed with RNA sequencing (Figure 1).

### 3.1. Nutritional Content of Fishmeal and Whey

The fishmeal used in this study contained 69.7% protein, while the whey contained 77.4% protein (Table 1). In addition, the fishmeal and whey contained 13.2% and 6.5% fat, respectively, while the whey contained 8% sugar (Table 1). As different amino acids have different effects on the postprandial metabolism, we compared the amino acid contents of the fishmeal and whey (Table 2).

The fishmeal contained twice as much arginine and 3.5-fold more glycine compared to whey. The high glycine content in fishmeal is likely due to the collagen present in salmon by-products, as approximately every third amino acid in collagen is a glycine (Gly-Xaa-Yaa)_n_, this amounts to, e.g., 29.3% glycine in salmon collagen alpha-1(I) (A0A1S3Q7E3_SALSA).

Whey contained more of the essential branched chain amino acids isoleucine (two-fold) and leucine (1.9-fold), as well as the non-essential amino acids glutamic acid, threonine, tryptophan and cysteine/cystine. The high content of essential amino acids (EAA) in whey has been described in numerous studies (reviewed in [24]), which has established whey as a so-called high-quality protein [25]. However, based on our analyses, fishmeal should also be classified as high-quality protein, as it contains all nine essential amino acids, and the fraction of EAA (g/100 g protein) is equal to or higher than the >24 g EAA/100 g protein that is recommend for adults by WHO [26]. Still, whey appears to be the better source of essential amino acids, with 38.2 g essential amino acids per 100 g whey, versus 24.5 g/100 g in fishmeal (Table 2).

Micronutrient analysis showed that salmon by-products could serve as a dietary source of certain vitamins and minerals. On average, we detected 10-fold higher levels of most the vitamins and minerals analysed in salmon fishmeal as compared to whey (Table S1). The salmon fishmeal contained high levels of cyanocobalamin (vitamin B12): 60.5 µg/100 g, corresponding to 4.5 µg in 30 capsules, i.e., 4.5 µg/dose. The corresponding value for whey was 0.9 µg/dose.

The participants therefore consumed twice as much as the recommended daily dose of 2 µg vitamin B12 when eating the fishmeal capsules (Nordic Nutrition Recommendations 2012 [6]). Fishmeal also contained high amounts of copper (8.6 mg/100 g; 0.64 mg/dose), zinc (140 mg/100 g; 10.5 mg/dose), and calcium (2500 mg/100 g; 187 mg/dose). Particularly for copper, the content was high, as compared to the recommended daily intake of 0.9 mg/day and the recommended upper intake level of 5 mg/day [6]. However, to reach the upper intake limit of copper, one would need to consume 58 g of fishmeal per day.

### 3.2. Effects of Intake of Fishmeal and Whey on Serum Amino Acids

**Figure 2:**
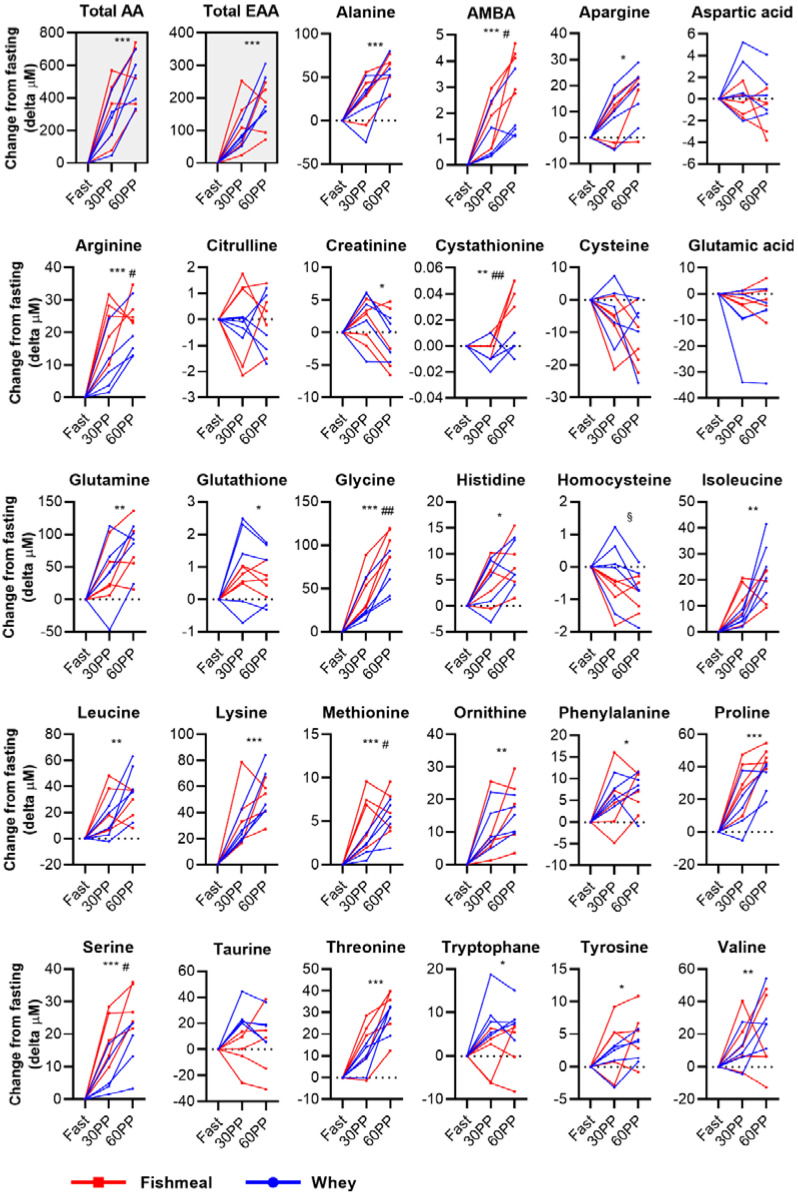
Changes in the serum amino acid concentration after the intake of fishmeal or whey. The concentration of 28 amino acids and amino acid derivatives was measured in serum with HPLC-MS/MS before (Fast), and 30 min postprandial (30PP) and 60 min postprandial (60PP), after the intake of fishmeal or whey. The total amino acids (Total AA) and total essential amino acids (Total EAA) were calculated as the molar sum of the individual amino acids measured in serum. Changes in the serum amino acids are expressed as change from fasting (delta μM). Red lines indicate each participant’s response to the intake of fishmeal, while blue lines indicate the response to whey. The overall difference between time points and protein sources was tested with a repeated measures, two-way ANOVA. * *p* < 0.05,** *p* < 0.01 and *** *p* < 0.001: overall difference between time points. # *p* < 0.05 and ## *p* < 0.01: overall difference between fishmeal and whey. § *p* < 0.05: interaction effect (time and protein source).

The intake of protein is expected to increase the serum levels of amino acids, and most, if not all, amino acid concentrations peak 30–60 min after a protein-rich meal [27,28,29,30]. We therefore measured the concentration of 28 different amino acids and amino acid derivatives in serum 30 and 60 min postprandially using HPLC-MS/MS (Figure 2). After the intake of fishmeal, the serum amino acid concentration increased by 331 μM (9.3%; *p* = 0.02) after 30 min and by 531 μM (15.3%; *p* = 0.003) after 60 min (Figure 2 and Figure S1). After the intake of whey, the total amino acid concentration in serum increased by 252 μM (7.7%; *p* = 0.02) and by 513 μM (15.6%; *p* = 0.001) after 30 and 60 min, respectively.

We found that 22 out of 28 amino acids/amino acid derivatives increased significantly after the intake of protein (Figure 2 and Figure S1). Most of these amino acids reached the highest concentration after 60 min, while some seemed to peak at 30 min, in line with what has been reported earlier [27,28,29,30]. While no differences in the total amino acid or total essential amino acid concentration were observed between the intake of fishmeal and whey, the difference in the postprandial response was significant for cystathionine and 2-aminobutyric acid (AMBA) and for the four amino acids arginine, glycine, methionine and serine, which all appeared to give a larger or faster increase with fishmeal as compared to whey (Figure 2).

Based on our results, we concluded that consumption of 5.2 g of protein from either whey or fishmeal was enough to significantly increase the concentration of most of the amino acids measured. In addition to protein, the capsules contained small amounts (<1 g) of sugar or fat; however, whether this was sufficient to influence the absorption or metabolic postprandial response was not addressed in our study. Fishmeal contained high levels of the amino acids glycine and arginine (Table 2), which may explain the increased postprandial serum concentrations of these amino acids.

Fishmeal consumption also gave a higher increase in serine, but this cannot be explained by differences in amino acid concentration, as the content of serine was slightly higher in whey compared with in fishmeal. The more prominent increase in cystathionine and AMBA following fishmeal ingestion could be explained by the rise in methionine levels. Fishmeal contains more methionine than whey, and it has been suggested that food-derived methionine increases both cystathionine and homocysteine, as well as methionine itself within 1–2 h [31]. While cystathionine appears to be an early and sensitive marker of changes in the flux through the transmethylation-transsulfuration pathway [31], AMBA, a by-product of cysteine biosynthesis from cystathionine, is suggested to reflect glutathione compensation against oxidative stress [32].

Previous studies have shown that different types of dietary protein are digested and absorbed at different rates. For instance, amino acids from whey are absorbed faster than from casein, which is less soluble and likely to slow gastric emptying [33]. Although we have not measured the absorption rates per se, our data indicate that the fishmeal protein was well absorbed, with an increase in the total serum amino acid concentration that was similar to whey (Figure 2). To our knowledge, no studies have examined the absorption of salmon fishmeal.

Absorption may be influenced by several factors, such as the denaturing effect of the fishmeal production process and the high amounts of insoluble proteins, such as collagen. Alcock et al. tested the absorption of several sources of collagen in healthy active males [30]. The intake of liquid collagen and gelatine resulted in a peak in the plasma total amino acid concentration after 30–60 min. Furthermore, the intake of collagen gave a substantially larger increase in glycine compared to the intake of various dairy protein supplements. This agrees with glycine being the most abundant amino acid in collagen by far, which again explains the enhanced glycine concentration after fishmeal consumption in our study.

### 3.3. Effects of Intake of Fishmeal and Whey on Metabolic Markers in Serum

As different sources of protein can influence postprandial insulin and glucose differently [34], we further measured the concentrations of glucose and insulin, as well as triglycerides, cholesterol, microCRP and the incretin GLP-1 in serum and plasma (Table 3). The intake of both fishmeal and whey gave small but statistically significant reductions in serum glucose and triglycerides after 30–60 min, while we did not observe any changes in the insulin, cholesterol, microCRP or GLP-1. Furthermore, there were no significant differences between the responses induced by the intake of fishmeal compared to whey (Table 3).

Although we did not observe any effects on insulin secretion in our study, amino acids are known to stimulate the secretion of insulin from β-cells, either directly or via the induction of incretins [35], both with and without the simultaneous ingestion of carbohydrates. The induction of insulin in response to amino acids is rapid; plasma insulin was found to reach a peak at around 15–30 min after the intake of 35 g casein in one study [36], whereas the intake of whey was found to induce the secretion of insulin from isolated islets 15 and 30 min after intake but not 45 min after intake [37].

However, the dose of amino acids given seems important, as the intake of 5 g of whey was found to be insufficient to induce insulin after 15 or 30 min [38]. It is therefore possible that the protein dose given in our study (5.2 g) was insufficient to induce the secretion of insulin. Cellulose, used as an excipient in the capsules, has been reported to modulate postprandial glycaemia and insulinaemia [38,39,40]; however, the total amount of cellulose from the 30 capsules (2.7–7.2 g) did not exceed the level believed to be needed to modify the glucose and insulin response [40]. Thus, our study appears well-suited to study the systemic effects of amino acids from two different protein sources without interference from insulin.

### 3.4. Intake of Fishmeal or Whey Did Not Influence Gene Expression in Liver Cells In Vitro

Having characterized the postprandial sera and established that the intake of fishmeal and whey give a differential rise in several amino acids, as well as changes in serum glucose and triglycerides, we went on to examine how the intake of fishmeal or whey influence gene expression in liver cells. We chose to focus on the liver due to its central role in coordinating the postprandial metabolism.

To test the potential effects on liver gene expression, we used a hepatic in vitro model, where we incubated cultured HepG2 cells for 6 h with 20% (*v*/*v*) of the individual sera collected in the postprandial study (Figure 1). In addition, control cells were incubated in serum-free medium. The incubation time was chosen based on the results from pilot experiments, where we measured the expression of 13 metabolic genes and calculated the gene regulatory potential in cells that had been incubated with serum for 3, 6 or 24 h (Figure S2).

Twenty percent serum in cell culture media is a dose frequently used in studies testing the properties of human serum on cultured cells [41]. Lees et al. used a similar strategy to test the postprandial effects of hydrolysates from blue whiting, whey protein and non-essential amino acids on muscle cells [41]. They showed that the intake of proteins equivalent to 0.33 g/kg body gave increased concentrations of amino acids and insulin in serum. Furthermore, serum taken after the consumption of fish protein hydrolysate or whey induced a hypertrophic response in cultured myotubes (20% serum for 4 h), as measured by puromycin incorporation and myotube thickness [41].

**Figure 3:**
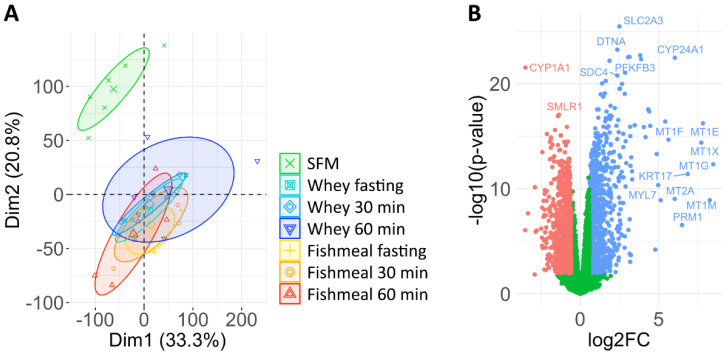
The gene expression profile of HepG2 cells incubated with or without human serum. (**A**) Principal component analysis of the gene expression (log_2_TPM) displaying the first two components. HepG2 cells were incubated with serum-free medium, medium supplemented with 20% human serum from fasting participants, or medium supplemented with serum taken 30 or 60 min after the intake of fishmeal or whey (*n* = 5–6 per condition; Figure 1). (**B**) Differential gene expression analysis of cells incubated with serum from fasting participants versus cells cultured in serum-free medium. Genes that were >1.5-fold up- or downregulated (fdr < 0.05) are indicated in blue or red, respectively. SFM: serum-free medium.

In our study, RNA expression was measured by RNA sequencing. To look for variations in the gene expression patterns between treatment groups, we first performed a principal component analysis (Figure 3A). We were unable to detect any clear clustering of samples incubated with the different human serum samples, while cells incubated with serum-free medium formed a separate cluster. We next performed pairwise, differential gene expression analyses between cells that were incubated with serum collected from fasting individuals or postprandial serum collected 30 or 60 min after the intake of fishmeal or whey.

We did not detect any genes that were differentially expressed (fdr < 0.05) after treatment with postprandial serum compared to fasting serum for any of the protein sources. We also compared the gene expression in cells incubated with serum after fishmeal or whey intake (30 min fishmeal vs. 30 min whey or 60 min fishmeal vs. 60 min whey). Again, we did not identify any differentially expressed genes (fdr < 0.05).

To ensure that the lack of differentially expressed genes in these analyses was not due to any systematic or methodological errors, we compared the gene expression in cells incubated with serum from fasting participants to control cells grown under serum-free conditions (Figure 3B). Here, we identified large transcriptomic differences. In total, 5471 of 17,061 transcripts were differentially expressed (fdr < 0.05). Of 12,702 protein coding genes, 1034 were more than 1.5-fold increased (fdr < 0.05), while 1419 mRNAs were more than 1.5-fold decreased (fdr < 0.05) with serum supplementation. The top 10 up- and downregulated genes that were highly expressed (TPM > 5) are listed in Table 4.

**Figure 4:**
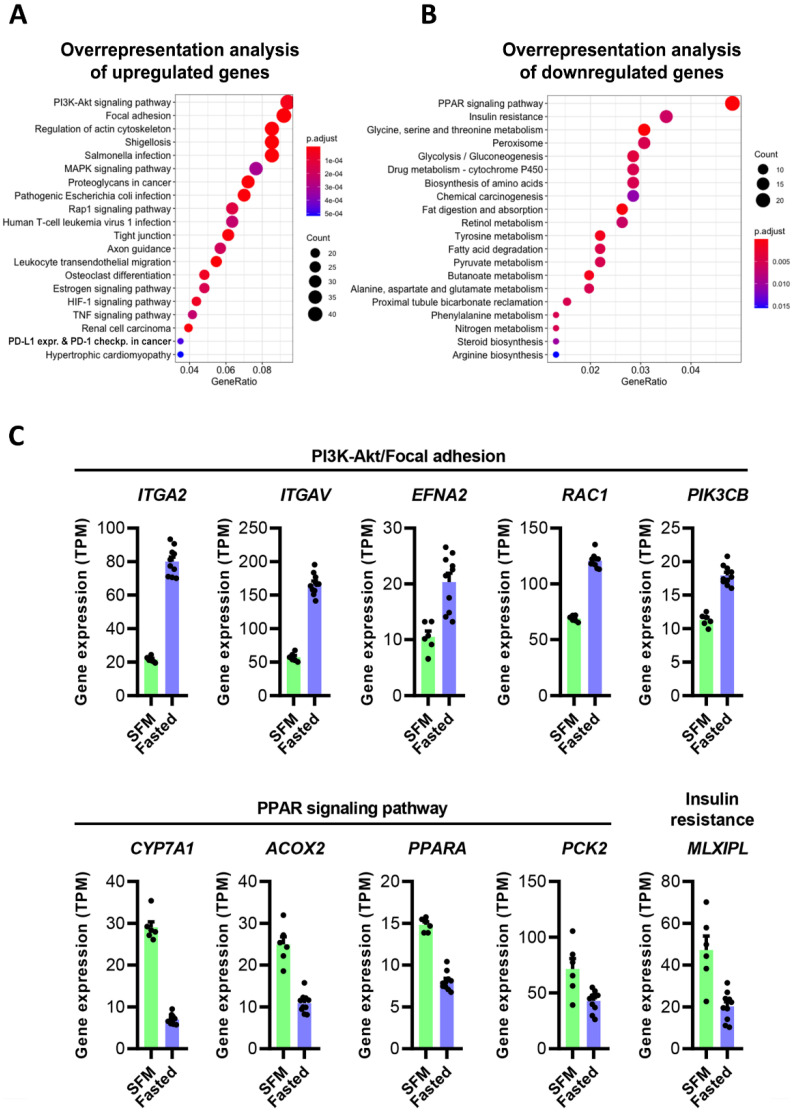
The gene expression in HepG2 cells exposed to human serum. (**A**,**B**) Overrepresentation analysis of upregulated or downregulated genes in cells incubated with serum from fasting participants, compared to cells in serum-free medium (>1.5-fold up- or downregulated, fdr < 0.05). (**C**) **Upper panel**: Selected genes in the PI3K-Akt and/or focal adhesion pathway that were upregulated in cells incubated with human serum. **Lower panel**: Selected genes in the PPAR signalling or insulin resistance pathways that were downregulated in cells incubated with human serum. Gene expression values are displayed as TPM (mean ± SEM). SFM: serum-free medium.

All gene expression values, as well as all differentially expressed genes are listed in Table S2B,C. Pathway analyses of the genes regulated up or down in cells incubated with fasting serum, compared to cells cultured in serum-free conditions (FC > 1.5, fdr < 0.05), showed PI3K-Akt signalling, Focal adhesion, and Regulation of the actin cytoskeleton as the top three upregulated KEGG pathways (Figure 4A), while PPAR signalling and Insulin resistance were the most downregulated pathways (Figure 4B). Several of the differentially expressed genes (Figure 4C) are found in two or more of these pathways, thereby, linking nutritional status and metabolism with cell signalling and cell–cell contact.

### 3.5. Potential Nutritional Value of Fish Protein or By-Products

To our knowledge, no published studies have investigated the postprandial effects of fishmeal from salmon by-products. In our study, we compared the effects of a single dose of protein (5.2 g) from salmon fishmeal and whey. When given as capsules or tablets, it is challenging to achieve a higher dose. Although we observed few effects on metabolic markers and gene expression in liver cells, this clearly does not change the relevance of fish by-products as an alternative food source.

The salmon fishmeal that we tested in this study contained a high amount of protein and some marine fatty acids [13]. In addition, it contained high amounts of several minerals and vitamins, including vitamin B12 (Table S1). The fortification of human food products with salmon fishmeal can therefore increase the nutritional value. One example is a study where fortifying pasta with fish protein powder showed an increase in the anti-oxidant and phenolic content of the pasta when assessed through in vitro digestion [42].

However, fishmeal is known for its distinct odour and taste, making it challenging to conceal in food products. This is one of the reasons why we chose to administer it as capsules in our study. Thus, it is interesting to note that a study using bread products fortified with fishmeal from Red-tailed Brycon (Brycon cephalus), reported that bread containing up to 8% fishmeal received sensory acceptance better than or as good as bread without fishmeal [43].

In future studies, it will be useful to study both the postprandial and long-term effects of higher doses of salmon fishmeal—for instance, as a supplement in complex meals or fortifier of food products. Previous studies have investigated the intake of fish protein or fish protein hydrolysates before or during a meal. Cod protein hydrolysate consumed before breakfast gave a slight reduction in postprandial insulin response but no change in glucose or GLP-1 when compared to casein [44]. Participants consuming a meal containing cod versus beef had a lower energy intake later in the day, indicating a potential role of cod protein on satiety, despite no significant differences in hunger or satiety measurements [45].

Several human studies have tested the metabolic effects of long-term low-dose fish-protein supplementation, but the data are inconclusive [10,11,12,13]. We recently used the same fishmeal as reported herein in a randomized controlled study on 74 pre-diabetic participants [13]. The participants were supplemented with the same dose (giving a total of 5.2 g protein/day) for 8 weeks. The supplement was well tolerated but did not influence the glucose tolerance or other measured cardiometabolic risk factors compared to a placebo.

Our results agree with another randomized study by Hovland et al. [12] where supplementation with 2.5 g protein from salmon per day for 8 weeks did not improve glucose tolerance or the markers of glucose regulation and insulin sensitivity. However, supplementation with cod, herring and especially milk protein (casein/whey mix), showed signs of an improvement in glucose tolerance [12]. This partly mirrors other intervention studies that have reported beneficial effects of cod protein on the glucose metabolism [10,11] and LDL cholesterol [11] possibly suggesting favourable health effects of fish protein intake.

The Hovland-study used a lower daily dose of protein in their trial than we used in the current study and in our prior human intervention study (2.5 g/day vs. 5.2 g/day) [12,13]. Moreover, they did not address the acute, postprandial effects of their supplementation. By profiling the hepatic transcriptome, we hoped to discover postprandial effects on liver metabolism not necessarily relating to the primary outcomes of the Hovland and Hustad studies, i.e., glucose regulation [12,13]. This, however, did not happen.

### 3.6. Limitations

One limitation with the current study is the source of venous blood. As hepatic transport, metabolism, and detoxification clearly modulate the postprandial serum composition, the ideal choice would be to use blood from the portal vein. However, in humans, the portal vein can only be accessed intraoperatively [46,47], thus, making it impossible to combine with postprandial studies. Another limitation is the low number of participants and the relatively low dose administered, making is possible to miss small but biologically relevant changes, especially when analysing serum components. However, for transcriptome analyses in monoclonal cell cultures, the sample size is adequate, and the lack of differentially expressed genes in these data reflect the delicate changes in serum constituents following a single dose of fishmeal and whey.

## 4. Conclusions

In this study, we demonstrated that fishmeal from salmon by-products was well-tolerated and that intake led to an increased amino acid concentration in the serum up to an hour after intake. We further measured a slight decrease in the serum glucose and triglycerides after the intake of whey or fishmeal despite no apparent induction of insulin. Lastly, serum collected from humans after the intake of whey or fishmeal did not show effects in the gene expression in cultured HepG2 cells when compared to the fasting serum. More studies are needed to investigate the effects of higher doses of fishmeal or salmon by-products, particularly as a component in food products. By-products from fish may provide a significant source of nutrition, and utilization as a human food source could increase food production efficiency and sustainability.

## Figures and Tables

**Table 1:** Macronutrient content of the fishmeal and whey.

Macronutrients (g/100 g)	Fishmeal	Whey
Fat	13.2	6.5
Carbohydrates	-	8.0
Sugar	-	8.0
Protein	69.7	77.4

**Table 2:** Amino acid content of the fishmeal and whey.

Amino Acids (g/100 g)	Fishmeal	Whey
***Essential amino acids***		
Histidine	1.52	1.65
Isoleucine	2.48	4.85
Leucine	4.57	8.50
Lysine	4.63	7.39
Phenylalanine (total)	2.50	2.56
Threonine	2.92	5.54
Valine (total)	3.19	4.55
Tryptophan	0.86	1.51
Methionine	1.84	1.64
Total EAA *	24.51	38.19
***Non-essential amino acids***		
Alanine	3.93	3.88
Arginine (total)	4.01	1.98
Aspartic acid	5.84	8.44
Glutamic acid (total)	7.62	13.9
Glycine	5.08	1.45
Hydroxyproline	0.57	<0.05
Ornithine	0.07	<0.05
Proline (Total)	3.22	5.03
Serine (Total)	2.95	4.03
Tyrosine (Total)	2.04	2.32
Cysteine + Cystine	0.75	1.64

* Total content of essential amino acids (g/100 g).

**Table 3:** Concentrations of metabolic markers, GLP-1 and microCRP in blood.

	Fishmeal ^1^				Whey ^1^				Fishmeal vs. Whey
	Fasting	30 min	60 min	*p*-Value Time Eff ^2^	Fasting	30 min	60 min	*p*-Value Time Eff ^2^	*p*-Value ^3^
s-Glucose, mmol/L	4.82 (0.49)	4.58 (0.40)	4.52 (0.44)	0.01 *	4.70 (0.62)	4.38 (0.56)	4.42 (0.69)	0.04 *	0.69
s-Insulin, pmol/L	36.0 (13.6)	57.6 (32.4)	37.4 (18.8)	0.13	39.4 (24.3)	37.8 (17.0)	46.8 (32.2)	0.45	0.87
s-Triglyceride, mmol/L	0.86 (0.25)	0.79 (0.22)	0.80 (0.25)	0.02 *	0.93 (0.22)	0.90 (0.22)	0.89 (0.21)	0.03 *	0.54
s-Cholesterol, mmol/L	4.60 (1.07)	4.44 (1.00)	4.46 (1.12)	0.17	4.76 (1.36)	4.66 (1.31)	4.66 (1.27)	0.30	0.80
s-microCRP, mg/L	0.38 (0.11)	0.38 (0.08)	0.36 (0.09)	0.59	0.32 (0.08)	0.34 (0.09)	0.34 (0.09)	0.37	0.49
*p*-GLP-1, pmol/L	3.37 (1.58)	4.07 (1.02)	3.67 (0.71)	0.30	3.63 (1.58)	3.36 (1.48)	3.68 (1.79)	0.30	0.74

^1^ Data are presented as mean (SD). *p*-GLP-1: plasma GLP-1. The other constituents were measured in serum. ^2^ Statistical testing of postprandial time effects was done with a repeated measures ANOVA for fishmeal and whey separately. * *p* < 0.05. ^3^ Differences in postprandial effects between fishmeal and whey were analysed with a two-way, repeated measures ANOVA.

**Table 4:** The top-10 upregulated and downregulated genes.

Gene Symbol	Gene Name	Log_2_FC	FDR	TPM *
***Upregulated***				
*MT1G*	metallothionein 1G	8.5	5 × 10^−11^	133.0
*MT1M*	metallothionein 1M	8.3	4 × 10^−8^	5.0
*MT1E*	metallothionein 1E	7.8	2 × 10^−14^	110.7
*MT1X*	metallothionein 1X	7.7	7 × 10^−13^	29.7
*CYP24A1*	cytochrome P450 family 24 subfamily A member 1	6.0	9 × 10^−20^	12.4
*MT2A*	metallothionein 2A	6.0	3 × 10^−8^	173.8
*MT1F*	metallothionein 1F	5.6	4 × 10^−13^	21.3
*ACTA1*	actin alpha 1, skeletal muscle	4.9	6 × 10^−12^	67.3
*TAGLN*	transgelin	4.4	2 × 10^−15^	87.7
*FSTL3*	follistatin like 3	4.0	4 × 10^−14^	121.0
***Downregulated***				
*CYP1A1*	cytochrome P450 family 1 subfamily A member 1	−3.5	5 × 10^−19^	12.3
*CYP7A1*	cytochrome P450 family 7 subfamily A member 1	−2.1	2 × 10^−13^	10.3
*SLC39A10*	solute carrier family 39 member 10	−1.9	7 × 10^−13^	22.9
*CYP4F3*	cytochrome P450 family 4 subfamily F member 3	−1.9	3 × 10^−13^	9.5
*TMEM140*	transmembrane protein 140	−1.7	8 × 10^−10^	8.9
*SPTLC3*	serine palmitoyltransferase, long chain base subunit 3	−1.7	3 × 10^−11^	24.6
*PLPPR1*	phospholipid phosphatase related 1	−1.7	1 × 10^−11^	12.4
*LIPG*	lipase G, endothelial type	−1.6	8 × 10^−14^	5.9
*ITIH1*	inter-alpha-trypsin inhibitor heavy chain 1	−1.5	5 × 10^−7^	6.0
*CLDN19*	claudin 19	−1.5	9 × 10^−7^	5.7

The top-10 upregulated and downregulated genes, sorted by log_2_FC, in HepG2 cells incubated with fasting, human serum, compared to cells incubated in serum-free medium. Only genes with TPM > 5 were selected. * Average TPM across all samples.

## Data Availability

The RNA sequencing data reported in this article is available at NCBI GEO (accession number GSE178584).

## Supplementary Materials

The following supporting information can be downloaded at: https://www.mdpi.com/article/10.3390/nu14081593/s1, Figure S1: Serum amino acid concentration before and after the intake of fishmeal or whey; Figure S2: Pilot studies; Table S1: Micronutrient content of salmon fishmeal and whey; Table S2A: Sample information; Table S2B: Gene expression values, expressed as transcripts per million (TPM) for all samples; Table S2C: Differential gene expression analysis (EdgeR) of HepG2 cells treated with human serum from fasting participants versus cells in serum-free medium.
