# Supplementary material for: Postprandial Effects of Salmon Fishmeal and Whey on Metabolic Markers in Serum and Gene Expression in Liver Cells
Source: Nutrients. 2022 Apr 12;14(8):1593. doi: 10.3390/nu14081593 (PMC9027870; doi:10.3390/nu14081593)
Supplement: Supplementary file 1 [file nutrients-14-01593-s001.zip › Figure S1.pdf]

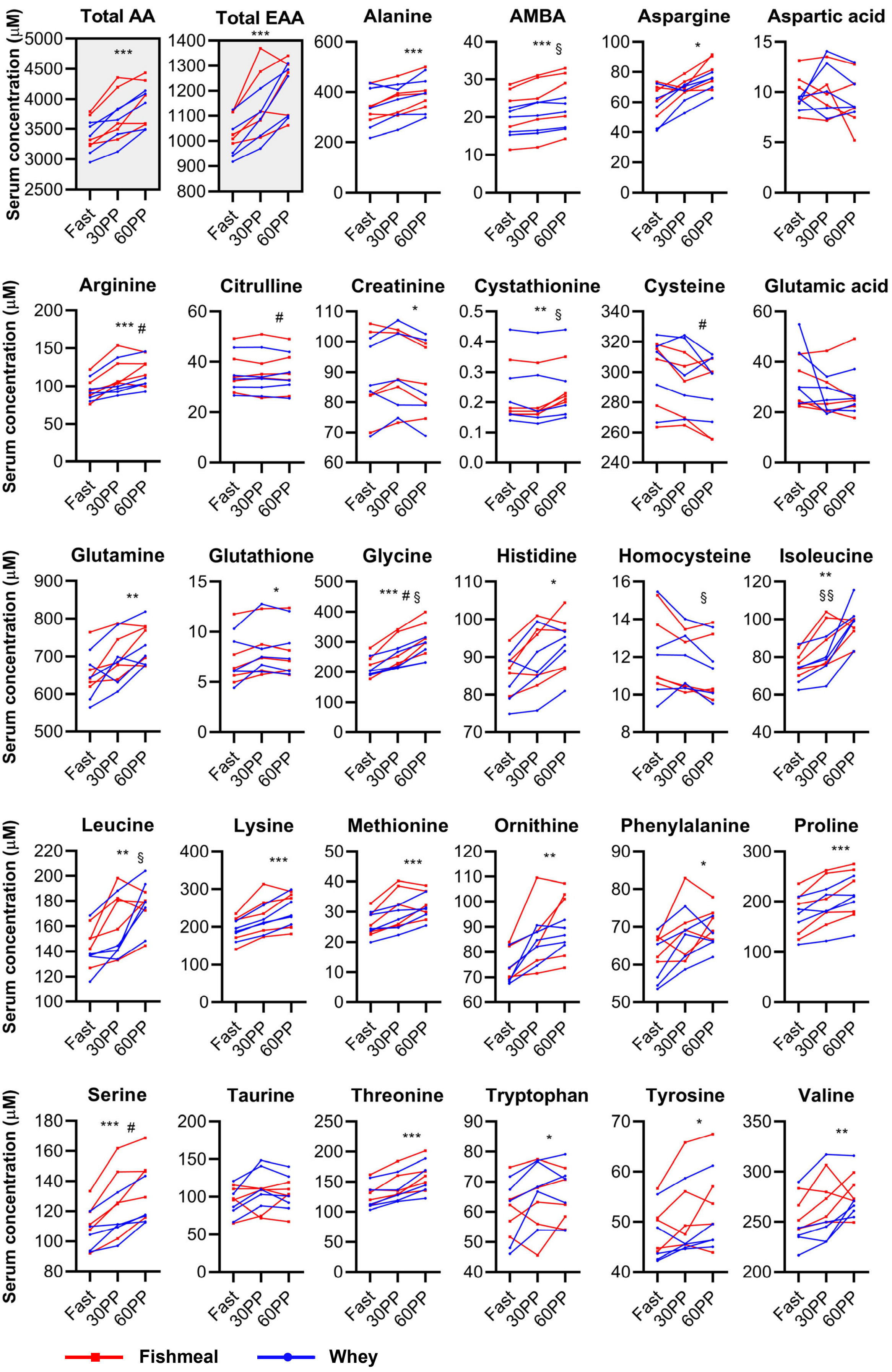

Figure S1. Serum amino acid concentration before and after intake of fishmeal or whey. The concentration of 28 amino acids and amino acid derivatives was measured in serum with HPLC-MS/MS before (fast) and 30 (30PP) and 60 min (60PP) after intake of fishmeal or whey. Total amino acids (Total AA) and total essential amino acids (Total EAA) were calculated as the molar sum of individual amino acids measured in serum. Red lines indicate each participant's response to intake of fishmeal, while blue lines indicate the response to whey. Overall differences between time points and protein source were tested with a repeated measures, two-way ANOVA. *$p<0.05$, **$p<0.01$, ***$p<0.001$: overall difference between time points. #$p<0.05$: overall difference between fishmeal and whey. §$p<0.05$, §§$p<0.01$: interaction effect (time and protein source).
