# Supplementary material for: Postprandial Effects of Salmon Fishmeal and Whey on Metabolic Markers in Serum and Gene Expression in Liver Cells
Source: Nutrients. 2022 Apr 12;14(8):1593. doi: 10.3390/nu14081593 (PMC9027870; doi:10.3390/nu14081593)
Supplement: Supplementary file 1 [file nutrients-14-01593-s001.zip › Figure S2.pdf]

A **B**

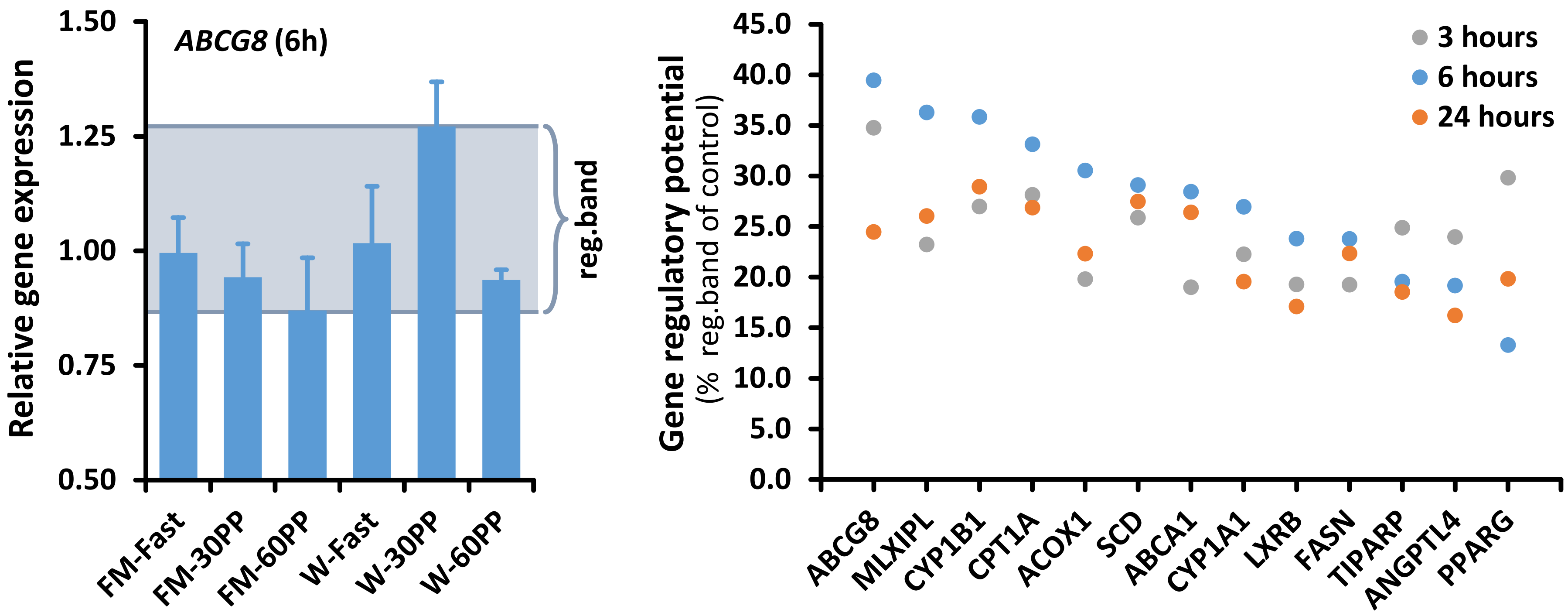

C

GENE	FORWARD PRIMER (5'-3')	REVERSE PRIMER (5'-3')
ABCG8	CCCGAGTCCTACGAAGATGC	CAGGTCTCGGAAGTCGTTGG
MLXIPL	CTACAAGAAGCGGCTCCGTA	AGCTGTTTGCACCATTGCTC
CYP1B1	CCTATGTCCTGGCCTTCCTTT	TGAGGAATAGTGACAGGCACAAA
CPT1A	CAGGAGACAGAGTTCCCTGG	TCTAACGTCACGAAGAACGCT
ACOX1	CTTCAACCCGGAGCTGCTTA	ATGTTCTCGATCTCTCGGCG
SCD	ACACCCAGCTGTCAAAGAGA	GCCAGGTTTGTAGTACCTCCTC
ABCA1	CGTTTCCGGGAAGTGTCCTA	GCTAGAGATGACAAGGAGGATGGA
CYP1A1	TGGTCTCCCTTCTCTACACTCTTGT	ATTTTCCCTATTACATTAAATCAATGGTTCT
LXRB	CCTGCCAGATGGACGCTTTC	CTTCAGAAAGGACGCACTGCT
FASN	CTTCAAGGAGCAAGGCGTGA	ACTGGTACAACGAGCGGATG
TIPARP	GGCAGATTTGAATGCCATGA	TGGACAGCCTTCGTAGTTGGT
ANGPTL4	TCCACCGACCTCCCGTTAG	GGCCACCTTGTGGAAGAGTT
PPARG	ACAGATCCAGTGGTTGCAGA	TCCACTTTGATTGCACTTTGGT
TBP	TTGTACCGCAGCTGCAAAAT	TATATTCGGCGTTTCGGGCA

Figure S2. Pilot studies. To determine the optimal incubation time HepG2 cells were incubated with serum from the postprandial study. Sera from all the participants (n = 5) were pooled at all time points/protein sources. **(A)** Gene expression from 13 metabolic genes was analysed by qPCR, normalized to *TBP* and the control group (W-Fast) set to 1. Data are presented as mean ± SEM (n = 2-3). The gene regulatory potential was calculated by defining the regulation band (global max – global min) for each gene, after 3h, 6h and 24h incubation and dividing that by the corresponding gene expression of W-Fast serum-stimulated cells. The graph showing the regulation of *ABCG8* expression in cells treated for 6h with pooled sera is included as an example. **(B)** Gene regulatory potential for the 13 metabolic genes after 3h, 6h and 24h serum incubation plotted in falling order based on the potential after 6 h incubation (blue dots). **(C)** Primer sequence for all primes used in these experiments.
